# Global Transcriptional Programs in Peripheral Nerve Endoneurium and DRG Are Resistant to the Onset of Type 1 Diabetic Neuropathy in *Ins2^Akita/+^* Mice

**DOI:** 10.1371/journal.pone.0010832

**Published:** 2010-05-26

**Authors:** Anne-Sophie de Preux Charles, Valérie Verdier, Jennifer Zenker, Bastian Peter, Jean-Jacques Médard, Thierry Kuntzer, Jacques S. Beckmann, Sven Bergmann, Roman Chrast

**Affiliations:** 1 Department of Medical Genetics, University of Lausanne, Lausanne, Switzerland; 2 Graduate Program in Neurosciences, University of Lausanne, Lausanne, Switzerland; 3 Swiss Institute of Bioinformatics, University of Lausanne, Lausanne, Switzerland; 4 Service of Neurology, Centre Hospitalier Universitaire Vaudois, Lausanne, Switzerland; 5 Service of Medical Genetics, Centre Hospitalier Universitaire Vaudois, Lausanne, Switzerland; Universidade Federal do Rio de Janeiro (UFRJ), Brazil

## Abstract

While the morphological and electrophysiological changes underlying diabetic peripheral neuropathy (DPN) are relatively well described, the involved molecular mechanisms remain poorly understood. In this study, we investigated whether phenotypic changes associated with early DPN are correlated with transcriptional alterations in the neuronal (dorsal root ganglia [DRG]) or the glial (endoneurium) compartments of the peripheral nerve. We used *Ins2^Akita/+^* mice to study transcriptional changes underlying the onset of DPN in type 1 diabetes mellitus (DM). Weight, blood glucose and motor nerve conduction velocity (MNCV) were measured in *Ins2^Akita/+^* and control mice during the first three months of life in order to determine the onset of DPN. Based on this phenotypic characterization, we performed gene expression profiling using sciatic nerve endoneurium and DRG isolated from pre-symptomatic and early symptomatic *Ins2^Akita/+^* mice and sex-matched littermate controls. Our phenotypic analysis of *Ins2^Akita/+^* mice revealed that DPN, as measured by reduced MNCV, is detectable in affected animals already one week after the onset of hyperglycemia. Surprisingly, the onset of DPN was not associated with any major persistent changes in gene expression profiles in either sciatic nerve endoneurium or DRG. Our data thus demonstrated that the transcriptional programs in both endoneurial and neuronal compartments of the peripheral nerve are relatively resistant to the onset of hyperglycemia and hypoinsulinemia suggesting that either minor transcriptional alterations or changes on the proteomic level are responsible for the functional deficits associated with the onset of DPN in type 1 DM.

## Introduction

Diabetic peripheral neuropathy (DPN) is the most common complication of diabetes mellitus (DM) [1]. Up to half of all individuals with DM develop DPN with a lifetime risk of lower extremity amputations estimated at 15% [2]. Typically, symptoms begin in the feet and progress to proximal regions, finally affecting the upper limbs, the abdomen and the thorax [3]. Diabetic patients may experience impaired tactile, thermal and pain sensations with dysautonomic manifestations such as sexual dysfunction, gastrointestinal abnormalities and cardiac arrhythmias. On the other hand, some patients also experience neural hyperexcitability inducing tingling, itching, burning, cramps and neuropathic pain [4], [5], [6]. A decrease in nerve conduction velocity (NCV) is the most consistent electrophysiological marker of DPN [4], [6]. Progression of DPN can be divided into 2 main phases [7]. The early stage of DPN is characterized by reversible deficits such as decreased NCV, decreased endoneurial blood flow, impairment of Na^+^/K^+^-ATPase and nitric oxide activities. Using animal models of type 1 DM (streptozotocin (STZ)-injected rats), it has been shown that these early defects cannot be attributed to nerve structural abnormalities [8]. At the later stage, other pathogenic components are progressively added, inducing structural changes such as segmental demyelination, axo-glial dysjunction and axonal atrophy. These structural modifications are irreversible [7].

Even though the above-mentioned pathological alterations affecting neuronal structures are relatively well described, molecular mechanisms underlying DPN remain poorly understood. Several interrelated metabolic abnormalities resulting from hyperglycemia, insulin and C-peptide deficiencies may be involved. So far, proposed mechanisms include altered metabolism of glucose via the polyol pathway, advanced glycation end products, increased protein kinase C (PKC) activity, altered neurotropism, perturbation of lipid metabolism, ischemia and reactive oxygen species [4], [6], [9], [10]. Currently, also the identity of the cell type first to be affected in DPN is unclear, as defects are observed in basically all cell types composing the nerve. Some authors claimed that DPN is primarily an axonal pathology [11], [12], whereas others support the hypothesis that DPN is a Schwann cell-related disorder [7], [13].

The search for changes in expression levels of candidate genes underlying DPN pathological hallmarks has been attempted previously, in either sciatic nerve or DRG compartments of the PNS [14]–[22]. These studies were complemented by more global transcriptomic approaches in either sympathetic ganglia [23], DRG [24] or in immortalized adult mouse Schwann cells [25]. However, the comparison of the effects of hyperglycemia on the stability of transcriptional programs *in vivo*, between the neuronal (DRG) and glial compartments of the peripheral nerve, was never attempted.

To address this question, we used *Ins2^Akita/+^* mice as a model of type 1 DM related DPN, and we concentrated on the early stages of DPN development when the pathological changes affecting neuronal function are still reversible. To define the progression of DPN in this model, we performed a detailed phenotypic characterization of *Ins2^Akita/+^* mice. NCV changes could be detected quite rapidly in this type 1 DM model, only one week after the onset of hyperglycemia. Based on these phenotypic data, we performed a gene expression profile of the sciatic nerve endoneurium (mostly containing Schwann cells) and DRG (mostly containing somas of sensory neurons) isolated from pre-symptomatic and early symptomatic *Ins2^Akita/+^* mice. We observed that both neuronal and glial transcriptomes are stable during early stages of diabetes. These data therefore suggest that the functional deficits associated with the onset of DPN are rather a consequence of a combination of minor transcriptional alterations and/or changes on the proteomic level.

## Results

### Phenotypic characterization of the development of DPN in *Ins2^Akita/+^* mice

We have selected *Ins2^Akita/+^* mice as a model of type 1 DPN. In this mouse model, diabetes is a consequence of an autosomal dominant mutation in the *Ins2* gene resulting in the improper folding of the Proinsulin 2 protein leading to hypoinsulinemia and hyperglycemia [26]. *Ins2^Akita/+^* mice develop multiple secondary complications associated with diabetes including neuropathy [27] and retinopathy [28].

**Figure 1:**
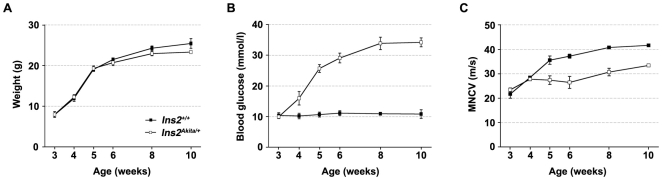
Phenotypic characterization of *Ins2^Akita/+^* mice. Body weight (**A**), tail vein blood glucose (**B**) and motor nerve conduction velocity (MNCV, **C**) were measured at depicted time-points in *Ins2^Akita/+^* and *Ins2^+/+^* mice. All results are expressed as the mean ± standard error of the mean (S.E.M.). (*Ins2^+/+^*: n = 4; *Ins2^Akita/+^*: n = 4).

**Figure 2:**
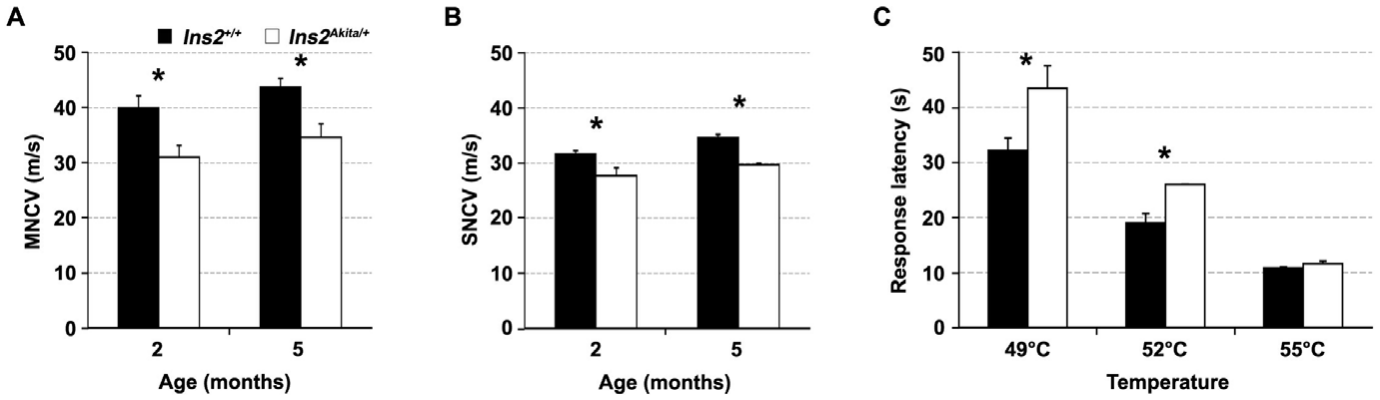
Characterization of motor and sensory behavior in *Ins2^Akita/+^* mice. Sciatic nerve motor (MNCV, A) and tail sensory (SNCV, B) nerve conduction velocities were compared in diabetic (*Ins2^Akita/+^*) and control (*Ins2^+/+^*) mice at two and five months of age. Results are expressed as the mean ± standard error of the mean (S.E.M). (*Ins2^+/+^*: n = 4; *Ins2^Akita/+^*: n = 4). C) Sensory performances were evaluated in two month old *Ins2^Akita/+^* and control littermates using the hot plate test at 49, 52 and 55°C. Results are expressed as the mean±standard error of the mean (S.E.M.) and analyzed by Student's t test (*Ins2^+/+^*: n = 8; *Ins2^Akita/+^*: n = 7). (*) p<0.05.

In order to select time-points for transcriptional analysis that are matching the onset of neuropathy in *Ins2^Akita/+^* mice, we first characterized the development of DPN in this model. Weight, tail vein blood glucose and motor nerve conduction velocity (MNCV) were followed in four male *Ins2^Akita/+^* and four control littermates between three and ten weeks of age. No significant variation of weight was observed between *Ins2^Akita/+^* mice and their control littermates (**Fig. 1A**). Blood glucose level started to increase in *Ins2^Akita/+^* between three to four weeks of age (**Fig. 1B**) and reached a plateau approximately five weeks after the onset of hyperglycemia. In order to match the onset of hyperglycemia with the onset of DPN, we measured MNCV in *Ins2^Akita/+^* mice and control littermates. Reduced NCV is an established early marker of DPN in patients [6]. At three and four weeks of age, no difference in MNCV was detected between *Ins2^Akita/+^* mice and control littermates. At five weeks of age, about one week after the onset of hyperglycemia, a decrease in MNCV was clearly present in *Ins2^Akita/+^* mice and this condition was maintained (but it did not worsen) as mice aged (**Fig. 1C**). In order to determine if the observed decrease in MNCV was also reproduced in sensory fibers, tail sensory NCV (SNCV) and MNCV were measured in two and five month old animals. SNCV could not be determined in younger mice, the tail being too small to allow reproducible results. In control animals, measured SNCV were lower than that of motor fibers, however the obtained values were in the range of the previously published results [27]. Similar to MNCV, the tail SNCV was decreased in *Ins2^Akita/+^* mice as compared to control littermates at two and five months of age, suggesting that both sensory and motor NCV are affected in this model of DPN (**Fig. 2A and B**). We have also observed a similar tendency for a decrease in SNCV at the level of the sciatic nerve (**Fig. S1**).

Sensory symptoms observed in DPN patients often include both loss of sensations and neuropathic pain [4], [29]. To evaluate *Ins2^Akita/+^* mice sensory behavior, the development of a thermal hypo- or hyper-sensitivity was assessed in two month old animals using a hotplate. In this test, *Ins2^Akita/+^* mice showed a significantly slower reaction time to heat at 49°C (*p* = 0.002) and 52°C (*p* = 0.005) (**Fig. 2C**). In contrast, no difference was observed at 55°C. These sensory symptoms were not associated with changes in intraepidermal nerve fiber density which is a marker of small fiber damage in more advanced human DPN (**Fig. S2**, [30]).

**Figure 3:**
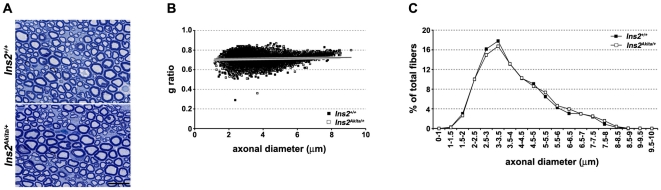
Morphometric evaluation of peripheral nervous system of *Ins2^Akita/+^* mice. **A**) Semi-thin toluidin blue stained cross sections of sciatic nerves from three months old *Ins2^Akita/+^* and control mice show well preserved nerve structures in both genotypes (scale bar: 15 µm). **B**) The scatter plot displays g ratios (g ratio = axon area/axon+myelin area) of individual axons as a function of the respective axonal diameters determined using sciatic nerves of three month old wild-type and *Ins2^Akita/+^* mice. Each point corresponds to one fiber. Thin dark-grey and thick light-grey lines represent the trend-lines for *Ins2^Akita/+^* and wild-type mice respectively. The two lines are superimposed reflecting close similarity of the two data sets. **C**) Axonal distribution represented as the percentage of axons for each class of sizes does not reveal any differences between wild-type and *Ins2^Akita/+^* mice.

The *Ins2^Akita/+^* animals presented a reduction of MNCV at five weeks of age. At this time-point, the peripheral nerves are still maturing as demonstrated by an increase in MNCV in wild-type animals between the ages of 5 and 10 weeks (**Fig. 1C**). In order to eliminate the possibility that the decreased MNCV present in *Ins2^Akita/+^* animals is a consequence of slowed PNS maturation leading to either hypomyelination and/or change in radial axonal growth (insulin signaling is known to affect Schwann cell and neuronal development [4], [5], [6]), we evaluated the level of myelination and axonal size distribution in three month old control and *Ins2^Akita/+^* animals. While at this developmental stage *Ins2^Akita/+^* animals presented decreased MNCV, SNCV and slower reaction time to heat (**Fig. 2**), we could not detect any structural changes in their PNS (**Fig. 3A–C**). Together our data therefore suggest that the DPN phenotypes observed in *Ins2^Akita/+^* animals during the early phase of the disease (the first two months after the onset) are a consequence of the effect of the diabetic condition on their PNS function and not on the structure.

### Analysis of the effect of the onset of diabetes on endoneurial and neuronal transcriptomes

**Figure 4:**
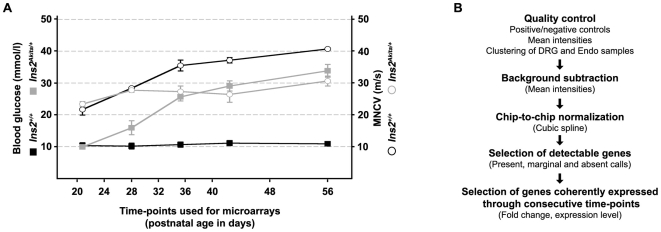
Design of the microarray experiment. **A**) Eight time-points were selected between 20 and 56 days of age. This time window covers the pre-symptomatic situation (P20, 20 days old), the onset of hyperglycemia (P24–P28), the onset of DPN (P32–P36) and later time-points with a clear symptomatic situation (P36–P56) in *Ins2^Akita/+^* mice. Squares: blood glucose; circles: MNCV. **B**) Schematic view of the microarray data analysis.

To detect the primary transcriptional changes associated with the development of DPN in *Ins2^Akita/+^* mice, gene expression profiling of the DRG and of the sciatic nerve endoneurium were carried out. Based on phenotypical characterization described above, eight time-points (postnatal days P20, P24, P28, P32, P36, P40, P48 and P56) covering the early stages of the neuropathy were selected (**Fig. 4A**). Until P24 (P20 and P24), *Ins2^Akita/+^* mice did not show any pathological phenotype (hyperglycemia or neuropathy). Starting from P28, glycemia was increased in affected mice and DPN was clearly detectable at P36, approximately one week after the onset of hyperglycemia. While hyperglycemia increased in *Ins2^Akita/+^* mice between P36 and P56, symptoms of DPN did not progressively worsen over time.

“Present”, “marginal” and “absent” calls were used for the first step of microarray data filtering (**Fig. 4B**). Since very closely spaced time-points were used, we considered all genes called “present” only at one developmental stage as artifacts and eliminated them from further analysis. Thus only probes called “present” in at least 2 consecutive time-points were selected leading to the elimination of approximately two thirds of the probes present on the chip (32′561 out of 48′318 total probes were eliminated in DRG arrays; 33′121 out of 48′318 total probes were eliminated in endoneurium arrays). As no chip replicate was included in our experiment we decided to filter the probes according to the coherence of their expression profiles. For this, we selected probes up- or down- regulated more than 3 or 1.5-fold in two or more consecutive time-points, with the additional constraint that the expression level of the selected probes should not vary over 1.5-fold outside these consecutive time-points (**Fig. S3**). This strategy aimed at selecting the most robust gene expression changes present through multiple time-points and helped us to efficiently eliminate the “zigzagging” expression profiles especially observed with genes expressed at low level (raw expression <100, see methods).

Using these filtering criteria, no probe showed a fold change greater than 3 in at least two consecutive time-points in either DRG or sciatic nerve endoneurium samples. Based on previously published studies (Price et al., 2006), we decided to also use a less stringent criterion of 1.5-fold change. In DRGs, 220 probes were selected using this threshold as being differentially regulated in at least 2 consecutive time-points (**Table 1**). However, the majority of these probes (146 out of 220) had a low expression level. Out of the 220 probes only 31 were up- or down-regulated in more than two consecutive time-points. In sciatic nerve endoneurium, 331 probes met our filtering criteria as being differentially regulated in at least two consecutive time-points (**Table 1**). As in DRG samples, most of these probes (267 out of 331) had a low expression level. Out of the 331 probes, 71 displayed a fold change above 1.5 in more than two consecutive time-points.

**Table 1:** Number of probes differentially expressed between *Ins2^Akita/+^* and *Ins2^+/+^* mice.

Tissue	# of consecutive time-points	# of probes observed
DRG	2	220 (12)
	3	31 (11)
	4	6 (5)
	5	3 (2)
	6	2 (1)
Sciatic nerve endoneurium	2	331 (8)
	3	71 (8)
	4	12 (5)
	5	2 (2)
	6	0

Only probes up- or down-regulated with a fold change of 1.5 in at least 2, 3, 4, 5 or 6 consecutive time-points and without any additional up- or down-regulation exceeding the 1.5-fold threshold were selected by our filtering criteria (see also Fig. S3). The number of genes tested by qPCR in each category is presented in brackets.

The lowering of the fold change threshold to 1.5-fold had as a consequence the decrease in the filtering stringency. In order to assess the confidence of the filtering procedure, we have examined some of the selected transcripts by quantitative PCR (qPCR, the list of tested transcripts is provided in **Table S1**). For both tissues, sets of probes differentially expressed in either 2, 3, 4, 5 or 6 consecutive time-points were selected. None of the 20 transcripts selected based on the 1.5-fold change criteria and evaluated by qPCR reproduced the expression pattern obtained with microarrays. All qPCR reactions designed for these confirmations resulted in PCR fragments of the expected size with good PCR efficiency. In addition, the specificity of all primers was confirmed by BLAST searching of the mouse genome database. Several housekeeping genes were used as normalizers, however using distinct normalizer genes did not affect the qPCR results. These different controls exclude PCR reaction problems as an explanation for the lack of reproducibility of our microarray results by qPCR.

Our analysis therefore showed that some expression profiles (mostly derived from genes with very low expression levels) pass our filtering criterion of at least 1.5 fold change in two or more consecutive time-points. However, the subsequent evaluation by qPCR revealed that the majority if not all of these candidates are false positives. Using more conservative filtering criteria (3 fold change in two or more consecutive time-points) we were unable to detect any transcriptional alterations in either endoneurial or neuronal compartments of diabetic mice.

### Effect of the onset of diabetes on the expression of genes playing a role in DRG neuron development and function

Since our expression profiling analysis did not reveal any global transcriptional changes in either the endoneurium or the DRG of diabetic mice, we decided to evaluate the expression of selected genes previously shown to play an important role in development or maintenance of these compartments.

**Figure 5:**
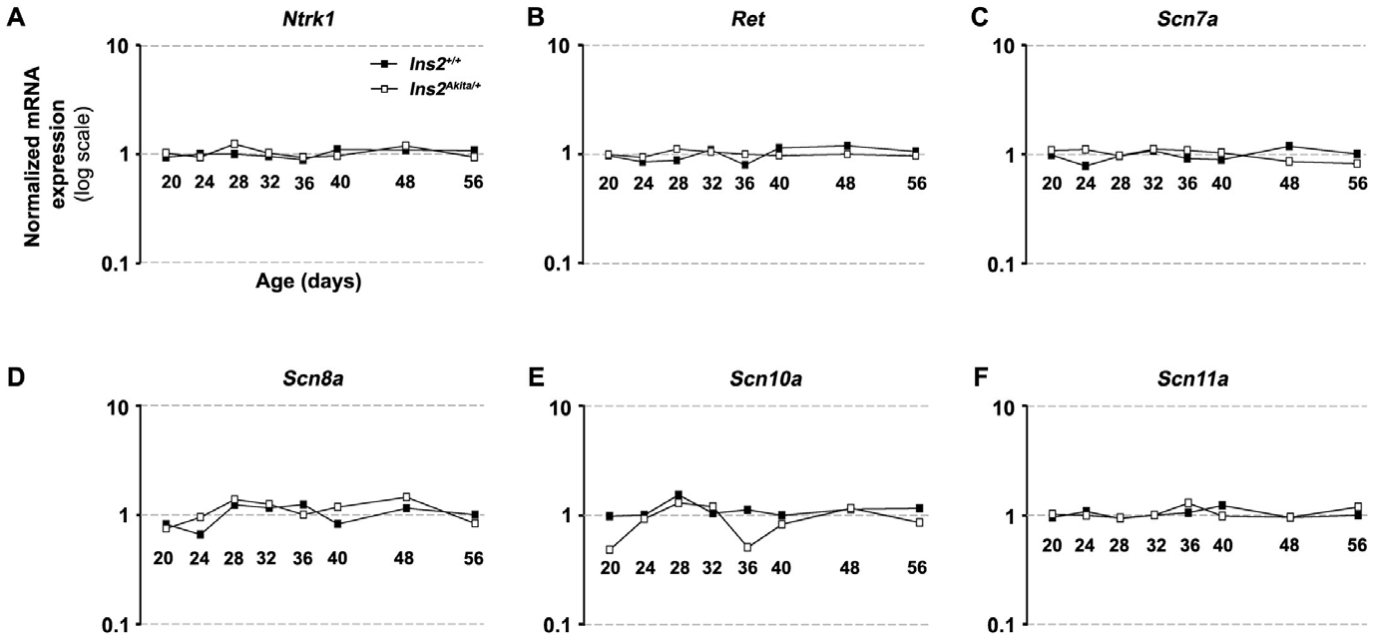
Expression profiles of selected genes involved in sensory neuron development and function. Normalized levels of expression at eight analyzed time-points (P20–P56) in both diabetic (*Ins2^Akita/+^*) and control (*Ins2^+/+^*) mice are shown. **A**) *Ntrk1*, **B**) *Ret*, **C**) *Scn7a*, **D**) *Scn8a*, **E**) *Scn10a*, **F**) *Scn11a*. All values were normalized by the median of the intensity obtained for a probe throughout the chips.

In DRG, the expression of sodium channels (crucial for normal propagation of action potentials) and of neurotrophic factors (involved in neuronal survival) was already studied [31], [32]. Recently, the developmental expression profiles of 17 sodium channels and neurotrophin associated genes had been reviewed [33]. These 17 genes were represented by 28 probes on the arrays that we used. Eight genes were previously shown to be expressed during early DRG development, but were absent in young adults (*Bdnf, Gdnf, Ngfa, Ngfb, Ngfg Ntf3, Nrtn* and *Scn3a* [33]). The expression of these genes was concordantly undetectable in our DRG samples. Nine genes were described to be expressed at adult stages with no major dynamic changes. Out of these 9 genes, six (*Ntrk1, Ret, Scn7a, Scn8a, Scna10a and Scn11a* [33]) were detected in our DRG samples (**Fig. 5**). None of these six genes met our less stringent filtering criterion of 1.5-fold change in two or more consecutive time-points, indicating that neither expression of sodium channels nor that of neurotrophin associated genes were affected by the onset of type 1 diabetes.

### Effect of the onset of diabetes on the expression of genes playing a role in Schwann cell myelination and myelin maintenance

**Figure 6:**
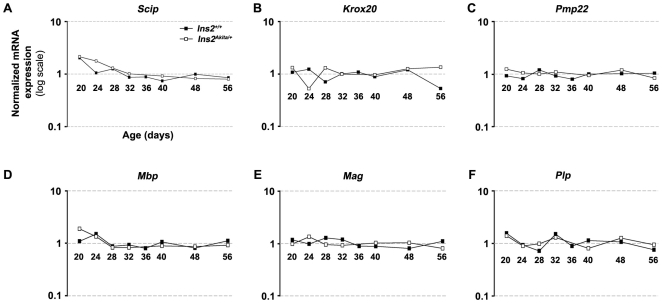
Expression profiles of selected genes involved in Schwann cell myelination. Normalized levels of expression at eight analyzed time-points (P20–P56) are shown for control (*Ins2^+/+^*) mice. In diabetic (*Ins2^Akita/+^*) mice, the expression was analyzed only at 7 time-points (data point P36 was excluded, see methods for more explanations). **A**) *Scip*, **B**) *Krox20*, **C**) *Pmp22*, **D**) *Mbp*, **E**) *Mag*, **F**) *Plp*. All values are normalized by the median of the intensity obtained for a probe throughout the chips.

Even though we could not detect any major modifications in myelin structure in *Ins2^Akita/+^* PNS (**Fig. 3A–C**), subtle molecular alteration in myelin gene expression could provide an early marker of later stage irreversible pathological changes. We have therefore analyzed the expression of genes encoding proteins involved in myelin synthesis and/or maintenance in the sciatic nerve endoneurium samples. Two transcription factors (Scip and Krox20) regulating progression of myelination and myelin protein expression respectively, together with the expression of four myelin proteins (*Pmp22, Mbp, Mag* and *Plp*) were evaluated. No difference in expression was observed between *Ins2^Akita/+^* and *Ins2^+/+^* mice (**Fig. 6**). *Scip* expression is known to peak during the onset of myelination (P2–P4) and then to decrease with time [34]. In our profiling, the decrease in the expression of this gene was accurately reproduced (**Fig. 6**). In addition, *Krox20, Pmp22, Mbp, Mag* and *Plp* showed no dynamic changes in adult stages, similar to previous studies [34]. The expression of cholesterol metabolism related genes was previously shown to closely match the expression of myelin genes [34]. None of the cholesterol metabolism related genes tested in our experiment met our filtering criterion of 1.5-fold change in two consecutive conditions (for example see *Cyp51, Dhcr7, Lss, Nsdhl, Sc4 mol, Sqle*; **Fig. S4**). Together these data indicate that the decreased NCV observed in DPN is not the consequence of significant changes in myelin gene expression that could potentially underlie structural defects of myelin observed in more chronic stages of DPN.

## Discussion

### Phenotypic characterization of the development of DPN in *Ins2^Akita/+^* mice

In the present study, we performed a detailed characterization of the onset of DPN in *Ins2^Akita/+^* mice. The *Ins2^Akita/+^* model provides the advantage of studying hyperglycemia without the confounding effect of toxic chemicals, such as streptozotocin, since *Ins2^Akita/+^* animals spontaneously develop hyperglycemia at approximately four weeks of age. Type 1 diabetes in the *Ins2^Akita/+^* mice is a consequence of an autosomal dominant mutation in the *Ins2* gene resulting in the improper folding of the Proinsulin 2 protein [26]. Transcription of the *Ins2* gene is normally responsible for the majority of the circulating insulin in the C57BL/6 wild-type mice. A recent report suggested that the intracellular accumulation of the misfolded Proinsulin 2 leads to progressive disruption of the insulin secretory pathway organelle architecture and thereby resulting in pancreatic β cell apoptosis [35].

Similarly to the previously described characterization of *Ins2^Akita/+^* mice [36] we found a progressive increase in blood glucose level starting between three and four weeks of age. Interestingly, our experimental design allowed us to detect the presence of a DPN much earlier than previously reported [27]. We observed a decrease in MNCV already one week after the onset of hyperglycemia and a decrease in SNCV at P56, one month after the onset of hyperglycemia, indicating that DPN affects both motor and sensory nerve fibers. In contrast, Choeiri and colleagues detected a decrease in SNCV in *Ins2^Akita/+^* mice only at four months of age. As the decrease in NCV of sensory fibers is smaller than in motor fibers, the sensitivity of measurement could be responsible for this discrepancy. A modest reduction in sensory and motor function was also observed in six month-old diabetic *Ins2^Akita/+^* mice [37].

The observed deficit in MNCV and SNCV in *Ins2^Akita/+^* mice does not seem to reflect a delay of PNS maturation since we could not detect any differences in either myelin thickness or axonal size in three month old affected animals and our microarray data does not reveal any changes in the expression of genes involved in either Schwann cell or neuronal maturation or function (see discussion below). This is in line with results obtained from analysis of the onset of DPN in STZ-induced type 1 diabetes at different developmental stages. If diabetes is induced in older animals, deficits in MNCV and SNCV appear and progress with a similar speed as compared to the induction of the disease in younger animals ([38] and discussion within).

In the hot plate test, *Ins2^Akita/+^* mice respond with increased latency to heat compared to the control littermates. These results are in line with the previously published evaluation of hind paw analgesia in older *Ins2^Akita/+^* mice, six months after the onset of diabetes [37].

Overall, our phenotypic characterization of *Ins2^Akita/+^* mice suggests that these mice develop DPN rapidly after the onset of diabetes and thus represent an interesting model of some of the DPN symptoms observed in patients which often present simultaneously positive (burning, neuropathic pain, abnormal sensation to temperature) and negative (numbness, injury insensitivity) sensory findings [4], [29].

### Transcriptional characterization of the development of DPN in *Ins2^Akita/+^* mice

We generated a time course of whole-genome expression data covering the pre-symptomatic situation, the onset of hyperglycemia, the onset of the DPN and finally the symptomatic period in *Ins2^Akita/+^* mice.

In sciatic nerve endoneurium samples, our gene expression analysis of myelin and cholesterol genes showed a good reproducibility with respect to previously published gene expression in wild-type mice [34]. Also, both myelin and cholesterol metabolism gene expression was not affected in *Ins2^Akita/+^* mice, thereby confirming previously observed stability of myelin structure even at a later developmental stage (48 weeks) in this model [36]. In DRG samples, we reproduced the previously described pattern of expression of sodium channels and neurotrophin associated genes [33]. The expression of these genes was not affected by the onset of diabetes in *Ins2^Akita/+^* mice. These results are concordant with previous observations in STZ-injected rats. Alterations of the expression of sodium channels Na_v_1.3, Na_v_1.6, Na_v_1.8 and Na_v_1.9 [19] or modifications of DRG structure [39] have been reported only in later stages of DPN.

The effect of diabetes on sympathetic neurons was previously comprehensively analyzed in STZ-injected rats after two and six weeks of diabetes [23]. Each of the two diabetes durations (2 and 6 weeks) was considered as a unique time-point. None of the 110 genes described as differentially expressed in para-vertebral or pre-vertebral ganglia in STZ-injected rats was up- or down-regulated with a fold change greater than 1.5 in two or more consecutive time-points in our experiment. Global expression changes have also been previously analyzed in STZ-injected rat DRG after one, four and eight weeks of diabetes [24]. In this study the authors chose a low threshold of 1.2 to maximize the number of differentially expressed genes (from 800 to 1200 per time-point). Similarly to our results, very small changes in gene expression were observed in diabetic DRG compared to control littermates. Out of the 113 published genes, none met our filtering criteria of 1.5-fold change in two or more consecutive time-points. These discrepancies are likely due to the fact that the onset of STZ-induced diabetes is more rapid than the progressive destruction of β-cells observed in *Ins2^Akita/+^* mice. STZ-injected rodents reach a very high level of glycemia within two days, whereas glycemia increases progressively in *Ins2^Akita/+^* mice.

DPN being a progressive disorder, we expected to find groups of transcripts progressively up- or down-regulated as hyperglycemia or DPN develops, with maximal changes in the later time-points. However, there was no correlation between phenotypic changes (onset of hyperglycemia and decreased MNCV) and transcriptional modifications in *Ins2^Akita/+^* mice. Using conservative filtering criteria of 3-fold change in two or more consecutive time-points, we were unable to detect any transcriptional alterations in either endoneurial or neuronal compartments of diabetic mice. By lowering the stringency criteria to 1.5-fold change in two or more consecutive time-points, we detected some potentially differentially expressed genes in either endoneurium or DRG of diabetic mice. However, none of the 20 expression profiles selected based on 1.5-fold change criteria could be confirmed by qPCR. These results indicate that based on our experimental design, the 1.5- fold threshold criterion is too low to reliably detect genes that are differentially expressed over the time-course.

The observed gene expression stability in both the sciatic nerve endoneurium and the DRG early after the onset of diabetes suggest that the early (reversible) phase of DPN is not a consequence of substantial transcriptional changes. We cannot however exclude the existence of transcriptional changes present only in a small fraction of cells in either the endoneurium or the DRG. The sciatic nerve endoneurium is mainly composed of Schwann cells, but also contains other cell types such as fibroblasts, macrophages and blood vessels. It is considered that approximately 10% of the nuclei in the sciatic nerve endoneurium belong to fibroblasts and another 2–9% to macrophages, while the remainder are Schwann cell nuclei [40]. Similarly to the endoneurium, DRG is composed of several populations of sensory neurons and other cell types such as satellite and Schwann cells [41], [42]. Thus, if transcriptional changes induced by diabetic conditions appear only in a specific cell population, these changes may be obscured by the messenger RNAs of the other cell types composing the tissues, which may prevent their detection by our current approach. We are working on new strategies that will allow us to efficiently purify mRNAs from selected endoneurial or DRG cell populations to test this hypothesis.

Together our data suggest that global transcriptional programs in both endoneurial and neuronal compartments of peripheral nerves are relatively resistant to the onset of type 1 DPN. The phenotypic changes observed during the onset of DPN, when the disease phenotypes are still reversible, are most probably a consequence of changes occurring at the post transcriptional level [9] or due to small changes in gene expression level or changes in restricted cell populations that are both below the level of sensitivity of the screen presented in this study. Major transcriptional changes may occur in the peripheral nervous system affected by DPN, but in more chronic stages when irreversible structural alterations appear.

## Materials and Methods

### Animals

All animals were housed in a controlled environment with a 12 h light/12 h dark cycle and free access to water and standard laboratory diet (except for fasting animals which had access only to water). Experiments were performed in accordance with the legal requirements of the University of Lausanne and the Canton of Vaud. C57BL/6J-Ins2^Akita^ (*Ins2^Akita/+^*) mice were obtained from The Jackson Laboratory (Bar Harbor, Maine, USA) and genotyped according to a previously described protocol [26].

### Measurement of blood glucose level

Tail vein blood glucose was determined with a glucometer Ascencia Contour (Bayer).

### Motor and sensory nerve conduction velocity (MNCV and SNCV)

All animals were anesthetized with a mixture of 10 µl/g of Ketanarkon 100 (1 mg/ml, Streuli) with 0,1% Rompun (Bayer) in PBS. For MNCV, the left and right sciatic nerves were stimulated at the sciatic notch and distally at the ankle via bipolar electrodes with supramaximal square-wave pulses (5 V) of 0.05 milliseconds. The latencies of the compound muscle action potentials were recorded by a bipolar electrode inserted between digits 2 and 3 of the hind paw and measured from the stimulus artifact to the onset of the negative M-wave deflection. MNCV was calculated by dividing the distance between the stimulating and recording electrode by the subtraction of the distal latency from the proximal latency. The sensory nerve action potential (SNAP) was recorded at the tail and sciatic nerve. For the tail recordings, the caudal nerve of the tail was stimulated distally and the bipolar electrode was inserted proximally at the base of the tail. Latency of the SNAPs was determined by measuring the stimulus artifact to the onset of the S-wave deflection. The SNCV of the sciatic nerve was recorded at the sciatic notch by stimulating the nerve with the bipolar electrodes placed in the dorsal part of the hindpaw. SNCV was calculated by dividing the distance between the stimulating and recording electrode by the latency. Results were expressed as the mean standard error of the mean (S.E.M.) and pair-wise comparisons performed using the Student's t-test.

### Morphometric analysis

Mice were perfused with 1% PFA and 2% glutaraldehyde in 0.1 M cacodylate buffer (pH 7.3) for 5 min. Sciatic nerves were dissected and post fixed by immersion in the fixative solution for 2 h at 4°C, washed in 0.1 M cacodylate buffer, and osmicated for 4 h in 1% OsO_4_ (Fluka). Nerves were rinsed in water, dehydrated, and embedded in epon 812 resin (Fluka). One-micrometer sections were stained with 1% toluidine blue and examined by light microscopy. Subsequent morphometric analyses were performed on micrographs using Image J plug-in (G ratio calculator) developed in collaboration with the cellular imaging facility of the University of Lausanne and available at http://cifweb.unil.ch.

### Intraepidermal nerve fiber (IENF) density quantification

Footpads from 10 week old male *Ins2^Akita/+^* and *Ins2^+/+^* mice were removed from the plantar area of the hind feet and fixed in Zamboni's fixative for 2 hours at room temperature. Tissues were washed three times in PBS, kept in 30% sucrose/PBS overnight at 4°C and subsequently embedded in OCT medium (Sakura). Frozen sections (50 µm) were prepared and six different sections (using every fourth section) from each animal were immunostained by a free-floating protocol. Sections were blocked in 0,3% Triton X-100 followed by an incubation with the goat anti-Collagen IV antibody (at a 1∶40 dilution; Millipore) for 24 h at 4°C diluted in blocking reagent. After three washes in PBS, samples were stained with the secondary antibody anti-goat Alexa 594 (at a 1∶200 dilution, Invitrogen) for 2 hours at room temperature. The sections were then stained with the rabbit anti-PGP 9.5 (at a 1∶400 dilution; Ultraclone) and anti-rabbit Alexa 488 antibody (at a 1∶200 dilution, Invitrogen). Finally, sections were transferred onto a slide in a drop of PBS and, after drying, mounted with Vectashield mounting medium containing DAPI to counterstain cell nuclei (Vector Laboratories). Four fields per section and three sections per animal were quantified for IENF density. Images based on the stack of consecutive 2 µm sections (usually 15 sections) were generated by using confocal microscopy (Leica SP5 AOBS Confocal Microscope). Single IENF crossing the basement membrane between dermis and epidermis were counted, whereas secondary branching and epidermal nerve fragments that do not cross the basement membrane were excluded from the quantification. The length of epidermis was measured using ImageJ and the linear density of IENF (IENF/mm) was obtained. Counting was carried out with the observer blinded to the origin (*Ins2^Akita/+^* and *Ins2^+/+^*) of the image.

### Sensory behavior assessment

All experiments were carried out by the same person, in the same laboratory and in the same environment. Testing started after habituation of the mice to the experimenter and to the environment. The development of thermal hypersensitivity was measured using a hot plate (Columbus Instruments). Mice were placed on a hot plate set at 49°, 52° or 55°C. The nociceptive behavior (paw licking or jumping from the hot plate) response latency was recorded and animals were removed immediately from the hotplate. Without response, animals were removed after 1 min, 45 or 20 seconds for the three temperatures respectively, to diminish the potential of thermal injury. Three trials separated by over ten minutes were averaged for each data point.

### Total RNA preparation

Sciatic nerve endoneuriums and L5 DRGs were dissected from P20, P24, P28, P32, P36, P40, P48 and P56 days old male *Ins2^Akita/+^* and *Ins2^+/+^* mice. Since we have previously shown that Schwann cells are sensitive to dietary changes [17], all animals were subjected to 14 h fasting and to a subsequent 6 h refeeding before sacrifice in order to limit the metabolic variation between individuals. For each group, tissues from five mice were pooled before RNA extraction. For each mouse, weight and blood glucose were measured (**Table S2**) to build groups that contained minimal phenotypic variation. In the set of animals used for microarray experiments, increased glycemia was initially observed at P24 and hyperglycemia became clear at P28, followed by a small decrease in weight at P48 and P56. Total RNA from sciatic nerve endoneuriums and DRGs was isolated using the Qiagen RNeasy lipid tissue kit (Qiagen) following the manufacturer's instructions. RNA quality was verified by agarose gel and/or by 2100 Bioanalyzer (Agilent) and the concentration was determined by the ND-1000 Spectrophotometer (NanoDrop).

### cRNA synthesis

For each condition (pool of five mice), 300 ng of total RNA was used to synthesize cRNA using the Illumina TotalPrep RNA amplification kit (Ambion) following the manufacturer's instructions. cRNA concentration was determined using a spectrophotometer, whereas cRNA quality was determined by 2100 Bioanalyzer (Agilent).

### Illumina arrays

The MouseWG-6 v1 expression Beadchips (Illumina) were used to determine differences in gene expression. Hybridizations were carried out using the Illumina gene expression system according to the manufacturer's instructions. Briefly, biotin-labelled cRNA (1.5 µg) was added to the array and incubated for 16–20 hours at 55°C. The bound biotin-labelled cRNA was then stained with streptavidin-Cy3. After hybridization, the microarray chip was washed, dried, and scanned by the Illumina BeadArray Reader. The absolute intensity of each probe on the image was generated with the BeadStudio software Version 1.5.1.3 (Illumina). Different controls were performed to assess the quality of the hybridization. The mean intensities of each chip, the mean intensities for negative or positive controls and the mean intensities obtained for random sequences (background level) were determined. All these mean intensities were within normal ranges and were also comparable among chips. Chips were also clustered based on their similarities. All chips hybridized with DRG samples were grouped within the same cluster. One of the chips hybridized with a sciatic nerve endoneurium sample (P36 *Ins2^Akita/+^*) was clustered within the group of DRG samples. This chip was therefore eliminated from the analysis. BeadStation 5003 data were extracted using the cubic spline normalization option of BeadStudio Version 1.5.1.3 (Illumina). Each tissue was normalized independently. Absent, marginal and present calls were defined according to the mean intensity level of the probes (BeadStudio). Data were then imported into GeneSpring V7.2 (Agilent Technologies) for data visualization and filtering. Raw intensities of present genes were ranging from 10 to 40′000. Genes represented by probes with a raw intensity under 100 (44% of probes scored as present in DRG samples; 47% of probes scored as present in sciatic nerve endoneurium samples) were therefore considered as low expressors. The difference in expression level between the 2 genotypes was calculated by dividing raw data obtained for *Ins2^Akita/+^* mice by raw data obtained for *Ins2^+/+^* mice. In this case, raw expression values less than 10 were converted to 10 (expression values <10 = “absent” calls). The array data is accessible through the ArrayExpress database (accession number: E-TABM-987; http://www.ebi.ac.uk/microarray-as/ae/).

### Quantitative RT-PCR

250–500 ng of total RNA was subjected to reverse transcription using the SuperScript™ III First-Strand Synthesis System for RT-PCR (Invitrogen) following the manufacturer's instructions. Resulting cDNA was used as a template for quantitative PCR (qPCR). The cycling conditions were 95°C for 10 min, followed by 40 cycles of 95°C for 15 s, and 60°C for 1 min. To detect and eliminate possible primer–dimer artifacts, a dissociation curve was generated by adding a cycle of 95°C for 15 s, 60°C for 1 min and 95°C for 15 s. All primer sets produced amplicons of the expected size and their specificity was also verified by BLAST searching of the mouse genome database. Sample quantitation was performed using a standard curve established from a serial dilution of a mix of the samples. Results were normalized using the reference genes cyclophilin or ubiquitin, which showed minimal changes in their level of expression in the array data. See **Table S1** for a complete list of primers used for qPCR.

## Supporting Information

Figure S1 Sciatic nerve SNCV measurements in three month old control and *Ins2^Akita/+^* mice. A tendency for reduced SNCV was observed in diabetic (*Ins2^Akita/+^*) mice (p = 0.12). Results are expressed as the mean ± standard error of the mean (S.E.M). (*Ins2^+/+^*: n = 4; *Ins2^Akita/+^*: n = 4).
(0.15 MB TIF)

Figure S2 Intraepidermal nerve fiber density assessment in 10 week old control and *Ins2^Akita/+^* mice. No difference was observed between control and diabetic (*Ins2^Akita/+^*) mice. Results are expressed as the mean±standard error of the mean (S.E.M). (*Ins2^+/+^*: n = 3; *Ins2^Akita/+^*: n = 3).
(0.15 MB TIF)

Figure S3 Schematic description of the microarray filtering criteria. Data are represented as a fold change between diabetic (*Ins2^Akita/+^*) and control (*Ins2^+/+^*) mice. A fold change of one represents an equal level of expression between *Ins2^Akita/+^* and *Ins2^+/+^* mice. Values above or below one represent, respectively, an increased or decreased level of expression in *Ins2^Akita/+^* mice. A cut-off of 1.5-fold change was used. In order for a probe to be selected by our filtering, fold change should therefore be >1.5 or <0.66 in 2, 3, 4, 5 or 6 consecutive time-points. In addition, we do not allow the probe to vary in the level of expression for more than 1.5-fold outside these consecutive time-points. Two examples of probes that would be selected using our criteria are shown: A) expression profile with an up-regulation in 2 consecutive time-points (black circles); B) expression profile with a down-regulation in 3 consecutive time-points. Two examples of probes that would be eliminated using our selection criteria are shown: C) expression profile with an up-regulation in two consecutive time-points (P28–P32), unchanged level of expression at P36–P40 and an up-regulation at P48 would be eliminated by our filtering; D) Expression profile with a down-regulation in 3 consecutive time-points (P28–P36) followed by an up-regulation in an additional time-point (P56) would also be eliminated by our filtering.
(0.59 MB TIF)

Figure S4 Expression of selected cholesterol metabolism related genes in sciatic nerve endoneurium samples. Normalized levels of expression at eight analyzed time-points (P20–P56) are shown for control (*Ins2^+/+^*) mice. In diabetic (*Ins2^Akita/+^*) mice the expression was analyzed only at 7 time-points (data point P36 was excluded, see methods for more explanations). A) Cyp51, B) Dhcr7, C) Lss, D) Nsdhl, E) Sc4 mol, F) Sqle. All values are normalized by the median of the intensity obtained for a probe throughout the chips.
(0.72 MB TIF)

Table S1 List of primers used for qPCR confirmations.
(2.73 MB TIF)

Table S2 Body weight and tail vein blood glucose measurements in *Ins2^Akita/+^* and control *Ins2^+/+^* mice used for the microarray experiment. Results represent the mean ± standard error of the mean (S.E.M.; *Ins2^+/+^*: n = 5; *Ins2^Akita/+^*: n = 5).
(1.24 MB TIF)
