# Supplementary figures and images for: Global Transcriptional Programs in Peripheral Nerve Endoneurium and DRG Are Resistant to the Onset of Type 1 Diabetic Neuropathy in Ins2Akita/+ Mice
Source: PLoS One. 2010 May 26;5(5):e10832. doi: 10.1371/journal.pone.0010832 (PMC2877074; doi:10.1371/journal.pone.0010832)

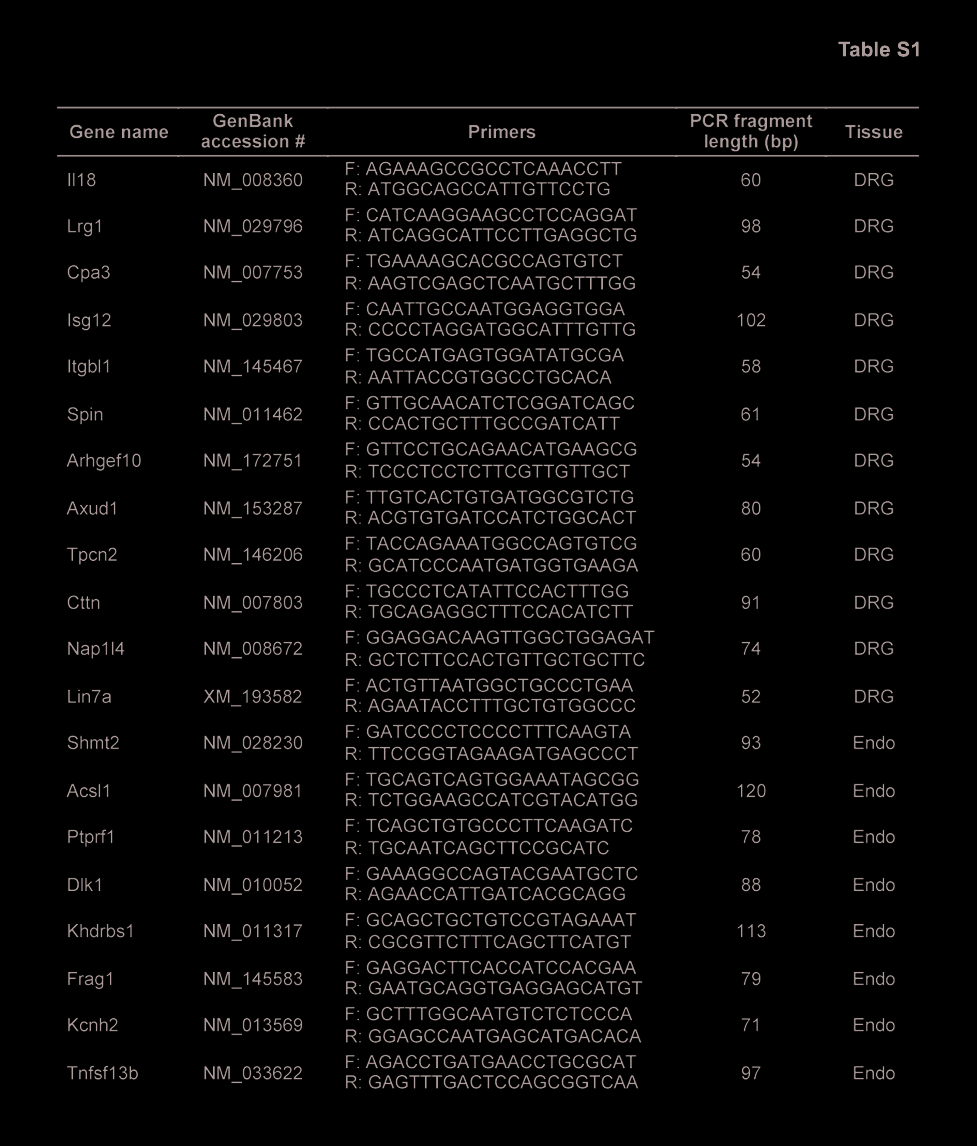

Supplement: Table S1 — List of primers used for qPCR confirmations. (2.73 MB TIF) [file pone.0010832.s005.tif]

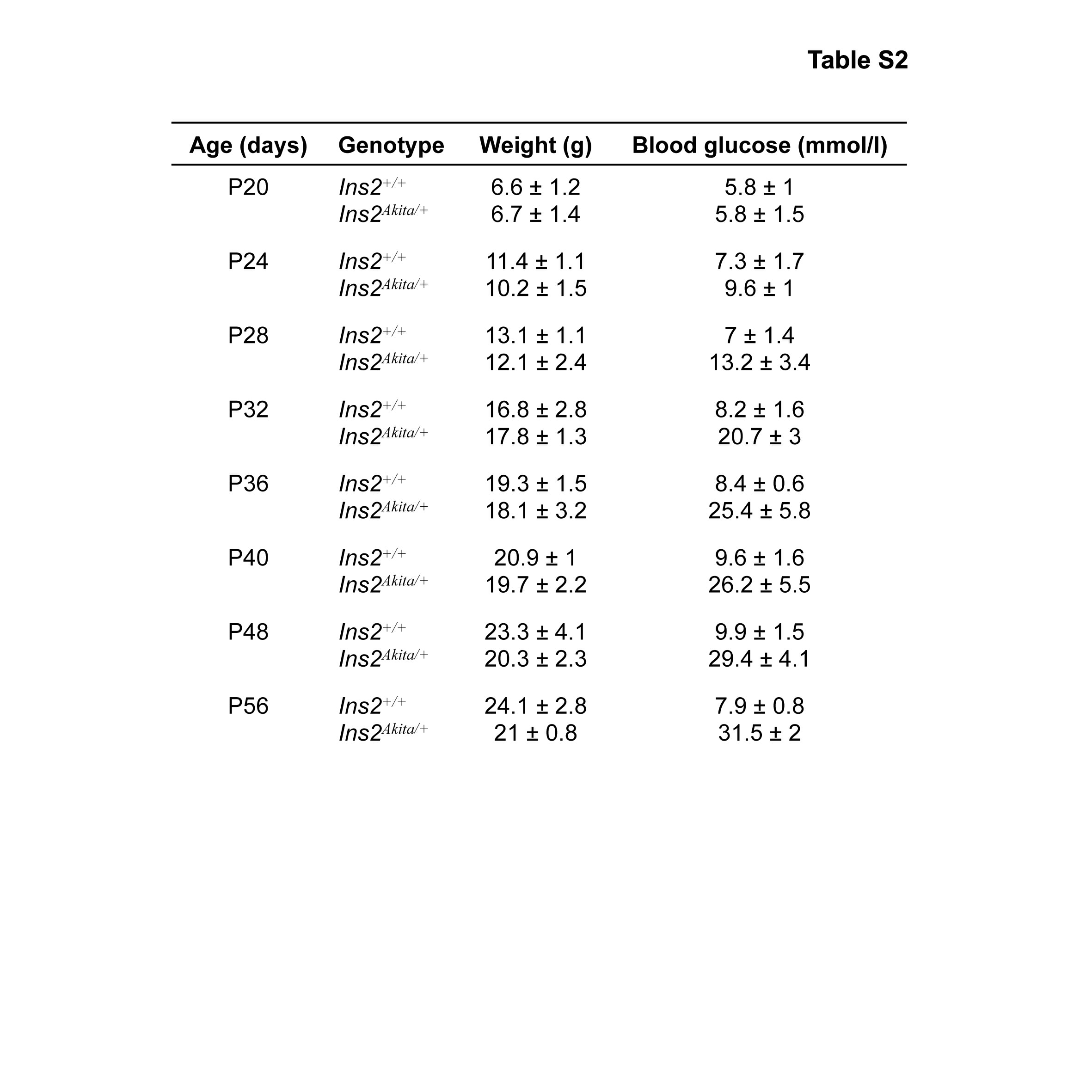

Supplement: Table S2 — Body weight and tail vein blood glucose measurements in Ins2Akita/+ and control Ins2+/+ mice used for the microarray experiment. Results represent the mean ± standard error of the mean (S.E.M.; Ins2+/+: n = 5; Ins2Akita/+: n = 5). (1.24 MB TIF) [file pone.0010832.s006.tif]
